# Practice guidelines for the molecular analysis of Prader-Willi and Angelman syndromes

**DOI:** 10.1186/1471-2350-11-70

**Published:** 2010-05-11

**Authors:** Simon C Ramsden, Jill Clayton-Smith, Rachael Birch, Karin Buiting

**Affiliations:** 1 National Genetics Reference Laboratory (Manchester), Saint Mary's Hospital, Hathersage Road, Manchester, M13 OJH, UK; 2 Department of Clinical Genetics, St. Mary's Hospital, Hathersage Road, Manchester M13 0JH, UK; 3 Department of Medical Genetics, Yorkhill NHS Trust, Yorkhill Hospital, Glasgow, G3 8SJ, UK; 4 Institut für Humangenetik, Universitätsklinikum Essen, Hufelandstrasse 55, D-45122 Essen, Germany

## Abstract

**Background:**

Prader-Willi syndrome (PWS) and Angelman syndrome (AS) are clinically distinct neurodevelopmental genetic disorders that map to 15q11-q13. The primary phenotypes are attributable to loss of expression of imprinted genes within this region which can arise by means of a number of mechanisms. The most sensitive single approach to diagnosing both PWS and AS is to study methylation patterns within 15q11-q13; however many techniques exist for this purpose. Given the diversity of techniques available, there is a need for consensus testing and reporting guidelines.

**Methods:**

Testing and reporting guidelines have been drawn up and agreed in accordance with the procedures of the UK Clinical Molecular Genetics Society and the European Molecular Genetics Quality Network.

**Results:**

A practical set of molecular genetic testing and reporting guidelines has been developed for these two disorders. In addition, advice is given on appropriate reporting policies, including advice on test sensitivity and recurrence risks. In considering test sensitivity, the possibility of differential diagnoses is discussed.

**Conclusion:**

An agreed set of practice guidelines has been developed for the diagnostic molecular genetic testing of PWS and AS.

## Clinical Background

Prader-Willi syndrome (PWS, OMIM ref. 176270) is characterised by severe hypotonia and feeding difficulties in early infancy, followed in later infancy or early childhood by excessive eating and gradual development of morbid obesity (unless eating is controlled by dietary restriction or behaviour modification). Motor milestones and language development are delayed. All individuals have some degree of cognitive impairment, although some will have an IQ within the normal range. A distinctive behavioral phenotype (with temper tantrums, stubbornness, manipulative behavior, and obsessive-compulsive characteristics) is common. Hypogonadism is present in both males and females, and manifests as genital hypoplasia, incomplete pubertal development, and in most, infertility. Short stature is common; characteristic facial features, strabismus, and scoliosis are often present, and non-insulin-dependent diabetes mellitus often occurs in obese individuals. Consensus diagnostic clinical criteria for PWS have been developed [1,2]; however confirmation of diagnosis requires genetic testing.

Angelman syndrome (AS, OMIM ref. 105830) is characterised by severe developmental delay, absent or severely limited speech, gait ataxia and/or tremulousness of the limbs, and a unique behavior with a happy demeanor that includes frequent and sometimes inappropriate laughter, smiling, and excitability. In addition, microcephaly and seizures are common. Affected individuals usually have a characteristic electroencephalography (EEG) appearance with striking high voltage activity. Developmental delay is first noted at around six months of age; however, the unique clinical features of AS may not manifest until after one year of age, and it can take several years before the correct clinical diagnosis is obvious. The diagnosis of AS rests upon a combination of clinical features as well as molecular genetic testing and/or cytogenetic analysis. Consensus clinical diagnostic criteria for AS have been developed [3,4].

## Genetic Background

**Figure 1:**
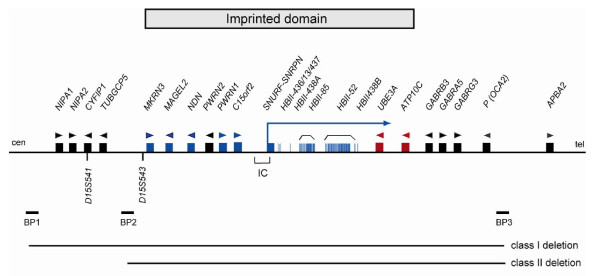
**Genes within the PWS and AS critical region**. Blue boxes represent paternally expressed genes; blue vertical lines, snoRNAs; red boxes, maternally expressed genes; black boxes, biallelically expressed genes and arrow heads represent the orientation of transcription. IC, imprinting centre; BP, common breakpoint cluster region.

The proximal long arm of human chromosome 15 (15q11-q13) contains a cluster of imprinted genes, which are under the control of a bipartite imprinting centre [5]. Some of these genes are expressed from the paternal or maternal chromosome only. PWS arises from the loss of function of paternally expressed 15q11-q13 genes as a result of either a paternally derived *de novo *deletion of this region, maternal uniparental disomy (UPD) of chromosome 15 or the silencing of the paternal alleles by an imprinting defect on the paternal chromosome. So far, several genes preferentially or exclusively expressed from the paternal chromosome have been described: *MKRN3, MAGEL2, NDN, PWRN1, C15orf2, SNURF-SNRPN *and several C/D box small nucleolar RNA (snoRNA) genes (see figure 1). At least two of these genes *SNRPN *and *NDN*, have differentially methylated CpG islands in their promoter regions that are methylated on the maternal chromosome leading to silencing of the maternal allele. Whereas most of the snoRNA genes are present as single genes (*SNORD64, SNORD107, SNORD108, SNORD109A *and *SNORD109B*), the two snoRNA genes *SNORD116 *and *SNORD115 *are present in 24 and 47 gene copies, respectively. It has been recently demonstrated that deficiency of *SNORD116 *(previously *HBII-85*) snoRNAs causes the key characteristics of the PWS phenotype [6,7], however one or more additional genes in the region are likely to contribute.

In AS the major disease mechanism is either a *de novo *maternally derived deletion of 15q11-q13, paternal UPD or an imprinting defect affecting the maternal chromosome. In addition point mutations in the E6-AP ubiquitin-protein ligase gene (*UBE3A*) are known to cause AS [8,9], Imprinted *UBE3A *expression is restricted to brain cells where expression is exclusively from the maternal chromosome and disruption of expression of this gene is now considered to be the major cause of the disease. There is one further gene in 15q11-q13 that is preferentially expressed from the maternal chromosome in brain and fibroblasts; *ATP10C *[10]. This gene is not expressed in AS patients with a deletion, uniparental disomy or an imprinting defect; however, its role in the disease is unclear. In contrast to PWS, approximately 10% of patients suspected of having AS have a genetic defect of unknown aetiology and alternative diagnoses are considered below.

Microdeletions in a small number of patients with PWS or AS have helped define an imprinting centre (IC), which has two critical regions, the AS-SRO (shortest region of deletion overlap) and the PWS-SRO [5]. By analysing a very large series of PWS and AS patients with an imprinting defect it has been shown that the vast majority of imprinting defects are primary epimutations that have occurred spontaneously in the absence of DNA sequence changes [11]. Furthermore, in approximately one third of patients with AS and a primary epimutation, the imprinting defect is present in a subset of cells only (somatic mosaicism), indicating that it occurred after fertilisation [12]. A summary of the causative genetic mechanisms and recurrence risks underlying these two diseases is given in tables 1 and 2.

**Table 1:** Molecular defects and recurrence risks in PWS.

Genetic defect	Proportion of cases	Recurrence risk
*De novo *deletion of 15q11-q13 on the paternal chromosome	75-80%	<1%
Maternal uniparental disomy (UPD) of chromosome 15	20-25%	<1%
Imprinting defects (with an imprinting centre deletion excluded)	≈1%	<1%
Imprinting centre deletion	≈ 10-15% of patients with an imprinting defect	Up to 50% (if present in father)

**Table 2:** Molecular defects and recurrence risks in AS.

Genetic defect	Proportion of cases	Recurrence risk
De novo deletion of 15q11-q13 on the maternal chromosome	70-75%	<1%
Paternal uniparental disomy (UPD) of chromosome 15	3-7%	<1%
Imprinting defect (with an imprinting centre deletion excluded)	2-3%	<1%
Deletions of the imprinting centre	≈ 10-15% of patients with an imprinting defect	Up to 50% (if present in mother)
*UBE3A *mutation	≈10%	50% if present in mother
No identifiable molecular abnormality	≈10%	Unknown (up to 50%)

There are a number of cytogenetic and molecular approaches to the confirmation of these two disorders. The most common is DNA-based methylation testing to detect abnormal parent-specific methylation within the PWS and AS critical region. This will detect more than 99% of individuals with PWS and approximately 80% of individuals with AS. *UBE3A *sequence analysis detects mutations in approximately a further 10% of individuals with AS, however *UBE3A *analysis is not considered further in this article.

## Methods

Current practice in the molecular analysis and reporting of PWS and AS was assessed by consideration of the external quality assessment returns submitted to the European Molecular Genetics Quality Network (EMQN) and the United Kingdom External Quality Assessment Scheme (UKNEQAS) over a five year period. The guidelines in this article were posted on the web-site of the UK Clinical Molecular Genetics Society (CMGS) for consultation and amendment between 19^th ^May, 2008 and 6^th ^January, 2010 and heads of the constituent laboratories were invited to comment. In the light of feedback amendments were made and the final document was ratified by the CMGS Executive Committee on 15^th ^January, 2010. In addition they were approved by the European Molecular Genetics Quality Network (EMQN) Steering Group on 22^nd ^January, 2010.

## Results

### Strategies for the analysis of PWS and AS

The approach to the laboratory diagnosis of AS and PWS depends on many factors, including the availability of samples, the arrangement of laboratory services and the patterns of referral. The most sensitive single approach to diagnosing PWS and AS is to study methylation patterns within 15q11-q13 using molecular genetic techniques. These will detect deletions, UPD and imprinting defects by establishing either a solely maternal methylated imprint (PWS) or paternal methylated imprint (AS). Broadly speaking, methylation studies take one of two forms:

(i) The detection of methylation status solely at the *SNRPN *locus by use of methylation specific PCR (MS-PCR) or Southern blot analysis. This approach will confirm a diagnosis but will provide no further information regarding the disease mechanism necessitating follow up studies (FISH and/or microsatellite analysis).

(ii) The simultaneous assessment of methylation status and genomic dosage at numerous sites across the 15q11-q13 region, by the use of methylation sensitive multiplex ligation-dependent probe amplification (MS-MLPA). This approach will confirm the diagnosis and further identify the presence of a causative deletion. However, in the absence of a deletion follow up studies (microsatellite analysis) are required to distinguish between UPD and an imprinting defect.

**Figure 2:**
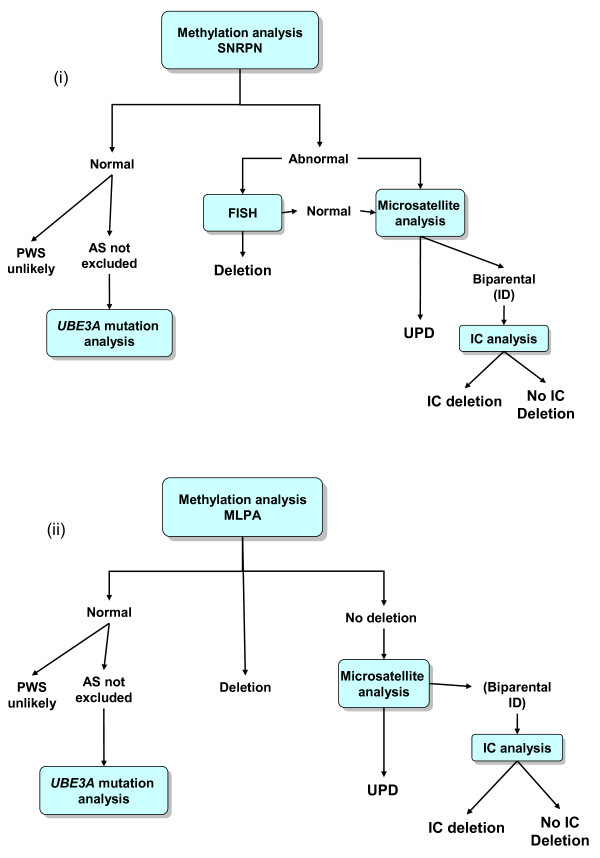
**Testing strategies for the molecular analysis of PWS and AS based upon (i) an initial methylation analysis at the SNRPN locus and (ii) an initial MLPA analysis**.

It is essential to note that approach (i) will not distinguish between the two classes of imprinting defect (ie. between one with no detectable deletion in the IC region and one due to a microdeletion of the IC). MS-MLPA however will give some limited information in this regard (see below). It is reasonable to request a standard karyotype analysis on the proband at the same time as a molecular analysis in order to investigate possible alternative explanations for the clinical presentation (if the PWS/AS investigations turn out to be negative). Upon the confirmation of a diagnosis, cytogenetic analysis of parental samples is recommended to investigate the possibility of balanced rearrangements, and genetic counseling should be offered to the family if the referral is not from a specialist in clinical genetics. Figure 2 shows an example of testing strategies for approaches (i) and (ii).

### Molecular genetic testing methods

#### MS-PCR

This is based on sodium bisulphite treatment of DNA followed by PCR using primers specific for differentially methylated sites within the *SNRPN *exon 1/promoter regions. Two approaches have been shown to work reliably in interlaboratory comparisons: Kubota *et al *[13] describe the use of two primer pairs that can be used separately (simplex PCR) or in combination (duplex PCR). However, it is strongly recommended that the primers are not used in a simplex reaction as this has been shown to result in spurious DNA amplification and/or misdiagnosis resulting from PCR failure [14]. Zeschnigk *et al *[15] describe an alternative assay with one common primer that anneals to both alleles and one specific primer each for the methylated and the unmethylated allele. In this case, the three primers are always used together in one reaction. The *SNRPN *exon 1/promoter region is highly conserved but contains a few very rare single nucleotide polymorphisms (SNPs) within the primer binding sites (see additional file 1). These changes have yet to be reported on current open access databases [K. Buiting personal communication].

#### Southern Blot Analysis

**Figure 3:**
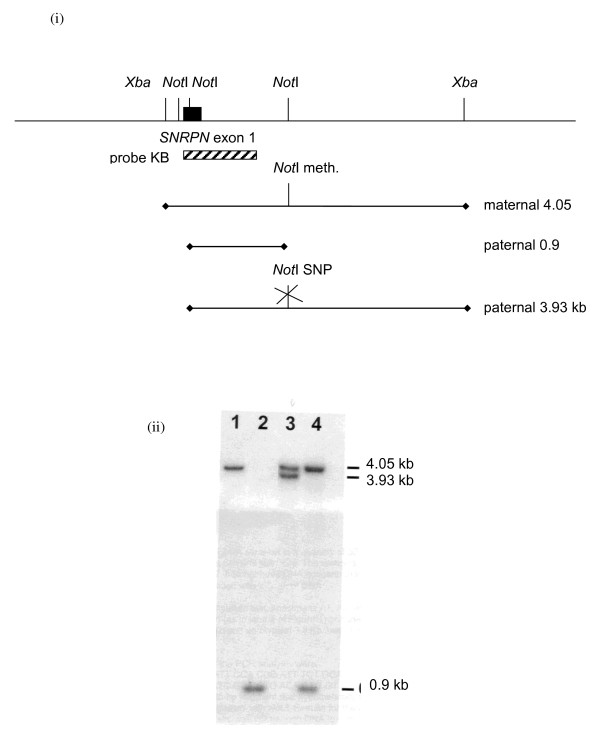
**The potential for mis-diagnosis as a result of a *Not *I restriction site polymorphism in *SNRPN *exon 1 (i) Genomic organisation**. (ii) Southern blot analysis using probe KB17 (lane 1 - PWS; lane 2 - AS; lane 3 - Normal (with *Not *I polymorphism); lane 4 - Normal).

The 15q11-q13 methylation status can be assayed by Southern blotting and a number of probes have been used in this regard. It is now considered essential that probes are chosen to assess methylation status at the *SNRPN *locus, rather than any other locus within the 15q11-q13 region. L48.25X, a 4.05 kb probe spanning exon 1 of the *SNRPN *gene detects the methylation at a C_p_G island within exon 1 of *SNRPN*. Genomic DNA digested with *Xba*I and the methylation-sensitive enzyme *Not*I shows 4.2 kb, 3.0 kb and 0.9 kb bands in unaffected individuals. The 4.2 kb band represents the methylated allele, which is absent in AS patients with a large deletion, paternal UPD or an imprinting defect. The 3.0 kb and the 0.9 kb bands represent the unmethylated allele which is absent in PWS patients with a large deletion, maternal UPD or an imprinting defect [16-18]. Whilst L48.25X requires pre-annealing, KB17 (a subclone of L48.25X) provides a useful alternative. Hybridisation with KB17 gives a 4.2 kb maternal and a 0.9 kb paternal band on *Xba*I and *Not*I digested DNA. It does not require pre-annealing, but is only 600 bp so can be easily washed off the filter in error. The final wash should be 2 x SSC at 65°C (for probe availability, please contact the authors). A rare restriction fragment length polymorphism affecting a *Not*I site in intron 1 can result in a 3.9 kb fragment for the unmethylated allele and can lead to false positive results in short run gels where the 4.05 and the 3.9 kb bands are not resolved (see figure 3).

#### Alternative techniques

A small number of laboratories use alternative methods of methylation analysis including PCR following restriction digestion with a methylation sensitive enzyme [19], melt-curve analysis [20] and pyrosequencing [21]. These techniques have not been widely adopted but have all been used successfully within a diagnostic context.

#### MS-MLPA

MS-MLPA provides a means to simultaneously detect copy number changes and DNA methylation within 15q11-q13 in a semi-quantitative manner. Most laboratories using this approach make use of a commercially available MS-MLPA kit available from MRC Holland http://www.mrc-holland.com. The ME028-B1 version of this kit contains 46 probes, 32 of which are specific for sequences in or close to the PWS/AS critical region on 15q11-q13. As a control for copy number changes, 14 probes outside the PWS/AS region are included. Among the PWS/AS specific probes, seven probes are methylation-sensitive and contain a *Hha*I restriction site. Five of the methylation sensitive probes from the PWS/AS region represent differentially methylated sites (four for the *SNURF-SNRPN *exon1/promoter region and intron 1 and one for the promoter region of *NDN*), one is from the completely unmethylated promoter regions of *UBE3A*, and another one is from the completely methylated *SNORD116 *snoRNA gene cluster region. The kit contains two additional methylation sensitive control probes for completely unmethylated sequences from other chromosomes that will indicate complete digestion by the *Hha*I enzyme.

Dosage analysis by MS-MLPA offers the opportunity to detect large deletions, the most frequent molecular lesions in patients with PWS and AS. In rare cases, a larger deletion can extend telemetric and include the probe for *APBA2*. Another advantage of the ME028-B1 MLPA kit is that small deletions affecting the *UBE3A *gene or the *SNORD116 *gene region can be detected.

MS-MLPA has become the method of choice in many diagnostic laboratories as it investigates methylation status at several loci, thereby reducing the risk of a false positive or false negative result due to SNPs; in addition, if one probe fails there are four remaining probes with which to assess methylation status.

MS-MLPA (ME028-B1) will also identify IC deletions in PWS and AS cases with an imprinting defect. The kit contains four probes for the *SNRPN *exon 1/promoter/intron 1, a region which represents the smallest region of deletion overlap in patients with PWS (PWS-SRO) and an IC deletion. Furthermore, two probes for *SNRPN *exon 3 and 7 are also included, which are deleted in most cases with an IC deletion. There are two probes which can be used to detect IC deletions in patients with AS and an imprinting defect. Both lie in the smallest region of deletion overlap found in patients with AS and an imprinting defect (AS-SRO).

There appears to be some naturally occurring variation in dosage and methylation status which must be taken into account when interpreting MS-MLPA results;

(i) For the two most centromeric probes, *NIPA1 *and *TUBGCP5*, copy number variation has been observed in healthy individuals. This has been attributed to deletions and duplications encompassing these two probes, and this complicates the definition of class 1 and class II deletions. This copy number variation has been observed in healthy individuals and seems to be non-pathogenic however it cannot be excluded as contributing to the phenotype in some individuals. Variation in the hybridization efficiency due to a copy number variation is also observed for the *SNRPN *exon *u1B *probe.

(ii) SNPs under hybridisation binding sites can influence probe signals. Consequently laboratories must exercise extreme caution when interpreting results from a single probe.

(iii) Normal variation in the degree of methylation at the *NDN *locus is frequently observed.

For prenatal diagnosis it should be noted that chorionic villi samples show a significant hypomethylation at the *NDN *locus and also for the *SNRD116 *probe (12273-L13798), which in blood DNA is completely methylated (Karin Buiting and Jasmin Beygo, personal communication). These results suggest that the methylation of the *NDN *locus is not fully established in chorionic tissue. It is therefore recommended that only the methylation status of the *SNRPN *locus is considered in the context of prenatal diagnosis.

It should be noted that the current commercially available MS-MLPA kits are not certified for diagnostic use and must be fully validated in individual laboratories prior to implementation. We recommend that recurrent variation observed in the MRC MS-MLPA kit is reported to the manufacturer to facilitate future kit development.

#### Microsatellite Analysis

When a diagnosis of AS or PWS is confirmed with any of the above techniques microsatellite analysis will often be required to distinguish between the various disease mechanisms. There are many microsatellites suitable for this purpose [22-27], and it is outside the scope of this article to provide a comprehensive list of suitable markers. However, it is worth noting the following markers that have been used widely in the past and are known to be compromised.

• *D15S113 *(LS6-1/2). This is a CA repeat from within the critical region. The presence of null alleles (or non-amplification alleles) have been observed with this marker and can complicate the analysis of AS and PWS cases. Under certain conditions, a non-amplification allele can be misinterpreted as a small deletion. The frequency of these alleles in families without AS has been estimated to be around 4%. Alternative primers can be designed; however, this marker is best avoided.

• *D15S817*. The presence of three alleles has been observed with this marker with certain primer sets due to complex duplications in the region where the marker is located. Since there is a low frequency/density of useful markers for the more centromeric PWS/AS region, the use of the following primer pair (D15S817F, 5'-TGGAACCAATAGGATAGACAC-3' D15S817R, 5'-GGTCAGCCTCCATAATCA-3') can resolve this problem.

Once the diagnosis of PWS or AS has been confirmed using methylation analysis, the interpretation of the microsatellite results is as follows:

##### a) Uniparental inheritance inside the critical region, biparental outside

In this case the disease is due to a deletion of the critical region. In rare cases, microsatellites may be used to confirm a smaller deletion within the critical region, however, laboratories must not interpret results from a single informative microsatellite without supporting evidence.

##### b) Uniparental inheritance both inside and outside the critical region

In this case, the disease is due to uniparental disomy. It is important to note that AS and PWS can be caused by either chromosomal isodisomy or heterodisomy. Further, heterodisomy or isodisomy at a single locus does not necessarily reflect the disomy status along the entire chromosome depending on the rate and level of crossing over and the meiotic stage at which non-segregation occurred.

##### c) Biparental inheritance both inside and outside the critical region

In this case the disease is presumed to be due to an imprinting defect.

### Interpretation of diagnostic testing results

#### Normal Methylation Result

A normal methylation pattern rules out PWS on the basis of most known cases to date and around 70-75% of AS cases. Despite early reports of possible deletion mosaicism in PWS [28,29] the case for deletion mosaicism remains unproven [30]. However in the case of the rare AS patients with a sporadic imprinting defect approximately one third appear mosaic for DNA methylation [11] and this group contains patients with an atypical phenotype that can overlap with PWS [31]. It is therefore essential that laboratories understand the limitations of a normal methylation result in PWS. In the case of AS, if the clinical suspicion remains high with normal methylation then it is recommended to undertake *UBE3A *analysis as mutations in this gene will have a recurrence risk of up to 50%, depending on the carrier status of the mother.

#### Deletions

The *de novo *interstitial deletion of chromosome 15, del(15)(q11-q13), which includes the entire imprinted domain plus several non-imprinted genes extends approximately 6 Mb and is found in the majority of patients with PWS and AS. In both syndromes, the same region is affected, but in PWS the deletion is always on the paternal chromosome, whereas in AS it is always on the maternal chromosome. The deletion occurs at a frequency of about 1/15,000 newborns and is probably one of the most common pathogenic deletions observed in humans. In a few patients, the region is deleted as the result of an unbalanced translocation. At the molecular level, two classes of deletions (class I and II) can be distinguished. In both classes, the distal breakpoints are close to, but telomeric to the *P *gene (breakpoint region 3, BP3, see figure 1). In class I deletions (30-40% of deletion cases), the proximal breakpoint is centromeric to the marker D15S541 (breakpoint region 1, BP1). In class II deletions (60-70% of deletion cases) the proximal breakpoint is between D15S541 and D15S543 (breakpoint region 2, BP2). The clustering of the deletion breakpoints is due to the presence of large duplicated sequence stretches of 200-400 kb in size in the common breakpoint regions that are susceptible to non-homologous crossovers [32,33]. Cases of *de novo *deletions should be further investigated by cytogenetic analysis to rule out the presence of (very rare) cytogenetic rearrangements in the appropriate parent (father for PWS and mother for AS) that may predispose to a deletion.

#### UPD

The second most frequent finding in PWS is maternal UPD (upd(15)mat) of chromosome 15. These patients have two maternal copies of chromosome 15 and lack a paternal copy. As the PWS genes are silent on the maternal chromosome, upd(15)mat is associated with a complete loss of function of these genes. The reciprocal finding is made in some patients with AS. These patients have two paternal copies of chromosome 15 and lack a maternal copy (upd(15)pat). In brain cells *UBE3A *is silent on the paternal chromosome, so upd(15)pat is associated with a complete loss of function of this gene in this tissue. Uniparental disomy arises in most cases of PWS as a result of a combination of meiotic and mitotic errors in female meiosis. During meoisis, the diploid set of chromosomes (n = 46) is reduced to a haploid set (n = 23). Nondisjunction of the homologous chromosomes 15 during female meiosis I or nondisjunction of the two sister chromatids during female meiosis II results in an oocyte with two chromosomes 15 or no chromosome 15. In these cases, fertilisation by a sperm with one chromosome 15 will result in a zygote which is trisomic or monosomic for chromosome 15 respectively. These conditions are not compatible with normal development, but can be 'rescued' by loss of one chromosome 15 from a trisomic cell or duplication of the paternal chromosome 15 in a monosomic cell. With trisomy rescue, in two-thirds of cases, one of the two maternal chromosomes will be lost from the trisomic cell, resulting in a normal set of chromosomes. If, however, the paternal chromosome is lost, the cell is left with two maternal chromosomes 15 (upd(15)mat). Duplication of the paternal chromosome 15 in a monosomic cell will lead to upd(15)pat.

Alternative mechanisms for UPD such as complementation involving both a nullisomic and disomic gamete or rescued paternal 15 trisomy have also been reported, however are considered rare [34].

#### Imprinting Defects

There are a small number of patients with either PWS (1%) or AS (2-4%) that show biparental inheritance for chromosome 15 markers, both inside and outside the critical region, but have an abnormal methylation pattern, characteristic for the syndrome. These patients are presumed to have an imprinting defect.

The majority of patients with an imprinting defect are sporadic cases without any detectable mutations in the IC region at the DNA sequence level. However in 10-15% of cases, the imprinting defect is caused by a microdeletion of the imprinting centre (IC). In most cases, the IC deletion is a familial mutation associated with a 50% recurrence risk, however in some cases the IC deletion is *de novo *or a consequence of germ line mosaicism in the father or the mother. In these families, the recurrence risk ranges from 0-50%, depending on the degree of the mosaicism in the germ line [11]. An IC deletion is the only kind of mutation found in patients with an imprinting defect with the exception of a single case where a familial inversion has been identified which disrupts the IC region [35].

Few laboratories are equipped to fully analyse these rare cases; however, it is important they are referred on to a specialist laboratory in order to confirm the nature of the imprinting defect and better understand the recurrence risk.

### Reporting

It is recommended that laboratories do not use joint PWS/AS report templates. It is essential to state clearly on the report the method(s) used to carry out genetic analysis, with an appropriate reference; for example, "Zeschnigk *et al. *1997 *Eur J Hum Genet*, **5**:94-99", or "ME028 MRC Holland http://www.mrc-holland.com)". It is essential that, where relevant, reports should inform as to the likelihood of recurrence, and, if the referral originates from a non-genetics specialist, genetic counselling is offered to all families where a diagnosis of AS or PWS is confirmed. In these cases cytogenetic analysis of the proband and father (for PWS) or mother (for AS) must be considered to rule out the remote possibility of cytogenetic rearrangements. More specific recommendations are as follows:

### Diagnostic referral for PWS (using either MS-PCR or Southern analysis)

(i) NORMAL RESULT. *Normal methylation result. This result excludes paternal deletion, uniparental disomy and an imprinting defect. The result makes a diagnosis of PWS highly unlikely*. The sensitivity of the test should be stated on the report.

(ii) DIAGNOSIS CONFIRMED: *Absence of paternal allele at 15q11-q13 by methylation analysis. This result confirms the diagnosis of PWS*. There is a common usage of terms such as "normal" and "abnormal" methylation pattern of the maternal or paternal allele/chromosome, however it is not appropriate to use these terms when the etiology is unknown. It is only appropriate to describe the imprinting pattern as "Abnormal" in patients with imprinting defect where it has been demonstrated that the paternal chromosome is aberrantly methylated. In deletion cases one allele is not present and therefore is not abnormally methylated. The same is true for maternal UPD, where the paternal allele is missing. With MS-PCR and Southern analysis it is best to describe what is observed; that is absence or presence of the relevant parental band and not simply to state that a methylation pattern typical of PWS is present. The laboratory should state that these approaches cannot determine the molecular cause of the result; and should request that parental blood samples be collected for microsatellite analysis in order to determine the mutational mechanism and recurrence risk.

### Diagnostic referral for PWS (MS-MLPA method)

(i) NORMAL RESULT: *Normal methylation and normal dosage at 15q11-q13. This result excludes paternal deletion, uniparental disomy and imprinting defect. A diagnosis of PWS is highly unlikely*. The sensitivity of the test should be mentioned on the report.

(ii) DIAGNOSIS CONFIRMED - DELETION IDENTIFIED: *Absence of paternal allele at 15q11-q13. This result confirms a diagnosis of PWS. The molecular cause of PWS is due to 15q11-q13 deletion*.

(iii) DIAGNOSIS CONFIRMED - NO DELETION IDENTIFIED: *Absence of a paternal allele at 15q11-q13 by MLPA, normal dosage at 15q11-q13. This result confirms the diagnosis of PWS. The molecular cause of PWS may be due to maternal UPD or an imprinting defect*. Laboratories should recommend microsatellite studies on the family to help confirm or exclude UPD.

(iv) DIAGNOSIS CONFIRMED - DELETION OF THE IMPRINTING CENTRE IDENTIFIED: *Absence of paternal allele for SNRPN exon 1/intron 1 probes and possibly SNRPN u1B probe. IC. deletion detected. This result confirms the diagnosis of PWS. IC deletions are associated with a recurrence risk of up to 50%*. In case of an IC deletion the father should be investigated for the presence of the deletion since familial IC deletion may have consequences for other family members.

### Diagnostic referral for AS (using either MS-PCR or Southern analysis)

(i) NORMAL RESULT: *Normal methylation. This result excludes maternal deletion, uniparental disomy and imprinting defects. The diagnosis of AS can not be confirmed in this patient. This result does not exclude a diagnosis of AS*. See also the comments regarding reporting "abnormal mathylation patterns" in PWS (above). It may be appropriate to offer *UBE3A *analysis after a clinical re-assessment of the patient.

(ii) DIAGNOSIS CONFIRMED: *Absence of maternal allele at 15q11-q13 by methylation analysis. This result confirms the diagnosis of AS. This may be due to a de novo deletion, uniparental disomy or an imprinting defect*. Collection of blood samples from the proband and parents is essential to determine the molecular cause and recurrence risk.

### Diagnostic referral for AS (MS-MLPA method)

(i) NORMAL RESULT: *Normal methylation and, normal dosage at 15q11-q13. This result excludes maternal deletion, uniparental disomy and imprinting defect. This result does not exclude a diagnosis of AS*. It may be appropriate to offer *UBE3A *analysis after a clinical re-assessment of the patient.

(ii) DIAGNOSIS CONFIRMED - DELETION IDENTIFIED: *Absence of maternal allele at 15q11-q13 by methylation analysis*. This *result confirms a diagnosis of AS. The molecular cause of AS is due to 15q11-q13 deletion*.

(iii) DIAGNOSIS CONFIRMED - NO DELETION IDENTIFIED: *Absence of maternal allele at 15q11-q13 by methylation analysis. This result confirms the diagnosis of AS. The molecular cause of AS may be due to paternal UPD or an imprinting defect*. Recommend microsatellite analysis to confirm/exclude UPD.

### Prenatal diagnosis

When the diagnosis has been confirmed and the molecular cause has been established in the index case, prenatal diagnosis can be performed. For cases of *de novo *deletions or disomy the recurrence risk is very low, however prenatal testing can be offered for reassurance. For cases of familial imprinting mutations due to an inherited deletion of the imprinting centre prenatal diagnosis should be offered as the recurrence risk is high (50%). Methylation status at *SNRPN *exon 1 is established early in embryonic development and testing DNA extracted from both amniotic cells and chorionic villi has been shown to give reliable results [36]. Fluorescent *in-situ *hybridisation (FISH) and/or microsatellite analysis can be used where appropriate to support the methylation results.

For cases of imprinting mutations due to deletions of the imprinting centre that are either *de novo *or a consequence of germ line mosaicism in the parent, the recurrence risk is difficult to predict but may be as high as 50%. In cases of imprinting defects with no detectable mutation, the recurrence risk appears to be low, however since the possibility of recurrence cannot be excluded a prenatal diagnosis should be offered. For these latter two scenarios prenatal diagnosis is performed by methylation analysis only.

### Differential diagnoses

#### Prader-Willi Syndrome

Diagnoses which need to be considered in infants with hypotonia include congenital myopathies, the congenital form of myotonic dystrophy and type 1 spinal muscular atrophy. Peroxisomal disorders should also be ruled out if chromosome 15 methylation is normal [37]. A further phenotype which presents with neonatal hypotonia and later onset obesity is attributable to deletions or epimutations of the *DLK1/GTL2 *locus on 14q32 as well as upd(14)mat [38,39].

When considering the differential diagnosis of older children with learning disability and obesity, Cohen syndrome, Borjesson-Forssman-Lehman syndrome (males), Bardet-Biedl syndrome and Alstrom syndrome along with chromosomal disorders including, diploid/triploid mosaicism and 1p36 microdeletion syndrome, should be considered [40].

#### Angelman Syndrome

Around 10% of patients with a clinical diagnosis of Angelman syndrome have no demonstrable abnormality at 15q11-q13 using the techniques described here. In rare cases, these patients may be mosaic for an imprinting defect. However, it is more likely that there is an alternative clinical diagnosis and a careful review of the patient's history, clinical features and EEG findings is recommended. One diagnosis which should be considered in girls is Rett syndrome (male Rett syndrome is rare but possible). It is extremely difficult to distinguish between Rett syndrome and AS during infancy when both can present with acquired microcephaly, ataxia and frequent smiling. Later, Rett syndrome may be distinguished by the presence of a history of developmental regression, the emergence of stereotypic hand-wringing movements, bouts of hyperventilation and the presence of vasomotor disturbance. If there is a very early onset of seizures, within the first few months, mutations within the *CDKL5 *gene should be considered [41]. Mowat-Wilson syndrome, caused by mutations in the *ZFHX1B *gene on chromosome 2, is associated with severe learning disability, limited speech, seizures and characteristic facial features that resemble those of AS. In addition Hirschsprung disease, congenital cardiac defects and agenesis of the corpus callosum may be associated with mutations in *ZFHX1B*. A strong indication is the characteristic appearance of the ear lobes which are upturned and look like "shell pasta". Pitt-Hopkins Syndrome (PHS) is a sporadic condition caused by mutations or deletions of the *TCF4 *gene on chromosome 18q; patients present with absent speech, seizures and facial features resembling AS, together with a sociable personality. The facial appearance in PHS coarsens with age and the lips in particular become prominent. Episodes of hyperventilation and apnoea may develop [42]. Recently, Zweier *et al*. reported several patients with autosomal recessive PHS caused by deletions or point mutations in the *NRXN1 *and *CTNAP2 *genes. Breathing anomalies, epilepsy and autistic features were prominent features in these cases [43]. An X-linked Angelman-like condition caused by mutations in the *SLC9A6 *gene has been reported [44]. Specific characteristics to look for in this condition are a slim body habitus and an unusually fast EEG rhythm. Several chromosome abnormalities have phenotypes that overlap with AS. The most common are the 1p36 subtelomeric deletion, a microdeletion of 17q21, and a terminal deletion of 22q13. Xq28 duplication including the *MECP2 *gene may also present with a phenotype suggestive of AS in males [45]. Profound neonatal hypotonia, the presence of constipation and Rett-like features distinguish Xq28 duplication from AS patients [46]. Recently several patients with 2q23.1 microdeletions encompassing the methyl binding domain gene *MBD5 *and a clinical and behavioural phenotype reminiscent of Angelman syndrome were reported [47]. Seizures, ataxia and sleep disturbance were common findings in this group of patients. Microarray studies are therefore clearly indicated in patients with AS-like features. Finally, some rare metabolic disorders may present with AS-like symptoms. Methyltetrahydrofolate reductase (*MTHFR*) deficiency and adenylosuccinate lyase deficiency have been reported as presenting with learning disbility, ataxia, seizures, autistic features and excessive laughter [48,49]. With MTHFR deficiency homocystinuria is present and treatment with folic acid and betaine may alleviate, though not completely cure symptoms.

## Discussion/conclusion

A practical set of molecular genetic testing guidelines has been developed for PWS and AS. In addition advice is given on appropriate reporting policies including advice on test sensitivity and recurrence risks. Feedback has been obtained from participants of the 2007 PWS/AS EMQN external quality assessment scheme (49 laboratories from 20 countries) and the heads of the constituent laboratories of the CMGS (46 laboratories from the UK). All comments received were minor; largely typographic corrections and some points of clarity. There was no disagreement on the recommendations made. All comments have been incorporated into this final document.

## Authors' contributions

All authors contributed equally to the content of this manuscript. SR drafted the manuscript and coordinated feedback in accordance with the procedures of the UK Clinical Molecular Genetics Society and the European Molecular Genetics Quality Network to ensure that consensus was agreed. All authors read and approved the final manuscript.

## Pre-publication history

The pre-publication history for this paper can be accessed here:

http://www.biomedcentral.com/1471-2350/11/70/prepub

## Supplementary Material

Additional file 1 Sequence of the *SNRPN *exon 1/promoter region before (upper black line) and after (lower red line) bisulphite treatment (chr.15; 22750953 - 22751602; Human Genome Browser; htg18). Single nucleotide polymorphisms (SNPs) in primer binding sites are boxed. In the bisulphite converted DNA sequence the X represents cytosines on methylated alleles. Primer binding sites for different PCR assays are shown as arrows. The binding site of two MLPA probes in the *SNRPN *exon 1/intron 1 region is given in blue. MF, forward primer for the maternal methylated allele; PF, forward primer for the paternal unmethylated allele; MR, reverse primer for the maternal methylated allele; PR, reverse primer for the unmethylated paternal allele. Click here for file
