# Supplementary material for: Practice guidelines for the molecular analysis of Prader-Willi and Angelman syndromes
Source: BMC Med Genet. 2010 May 11;11:70. doi: 10.1186/1471-2350-11-70 (PMC2877670; doi:10.1186/1471-2350-11-70)
Supplement: Additional file 1 — Sequence of the SNRPN exon 1/promoter region before (upper black line) and after (lower red line) bisulphite treatment (chr.15; 22750953 - 22751602; Human Genome Browser; htg18). Single nucleotide polymorphisms (SNPs) in primer binding sites are boxed. In the bisulphite converted DNA sequence the X represents cytosines on methylated alleles. Primer binding sites for different PCR assays are shown as arrows. The binding site of two MLPA probes in the SNRPN exon 1/intron 1 region is given in blue. MF, forward primer for the maternal methylated allele; PF, forward primer for the paternal unmethylated allele; MR, reverse primer for the maternal methylated allele; PR, reverse primer for the unmethylated paternal allele. [file 1471-2350-11-70-S1.DOC]

**Additional file 1:** Sequence of the *SNRPN* exon 1/promoter region before (upper black line) and after (lower red line) bisulphite treatment (chr.15; 22750953 – 22751602; Human Genome Browser; htg18). Single nucleotide polymorphisms (SNPs) in primer binding sites are boxed. In the bisulphite converted DNA sequence the X represents cytosines on methylated alleles. Primer binding sites for different PCR assays are shown as arrows. The binding site of two MLPA probes in the *SNRPN* exon 1/intron 1 region is given in blue. MF, forward primer for the maternal methylated allele; PF, forward primer for the paternal unmethylated allele; MR, reverse primer for the maternal methylated allele; PR, reverse primer for the unmethylated paternal allele.

TTTCTTTAGT ATTTTACACA TTTTAAAAAA CAGGTAGACA TGTCCATTGA TCCCAGGTTG

TTTTTTTAGT ATTTTATATA TTTTAAAAAA TAGGTAGATA TGTTTATTGA TTTTAGGTTG

CTTATGGTTT CTAGAGGCCC CCTCTCATTG CAACAGTGCT GTGGGGCCCT AGGGGTCCAG TTTATGGTTT TTAGAGGTTT TTTTTTATTG TAATAGTGTT GTGGGGTTTT AGGGGTTTAG

MLPA probe (11181-L11865)

TAGCCCCCTC CCCCCAGGTC ATTCCGGTGA GGGAGGGAGC TGGGACCCCT GCACTGCGGC

TAGTTTTTTT TTTTTAGGTT ATTTXGGTGA GGGAGGGAGT TGGGATTTTT GTATTGXGGT

C

AAACAAGCAC GCCTGCGCGG CCGCAGAGGC AGGCTGGCGC GCATGCTCAG GCGGGGATGT

AAATAAGTAX GTTTGXGXGG TXGTAGAGGT AGGTTGGXGX GTATGTTTAG GXGGGGATGT

Zeschnigk MF

Kosaki MF

Kubota PF

Kosaki PF

Kubota MF

MLPA probe (4106-L13905)

A

GTGCGAAGCC TGCCGCTGCT GCAGCGAGTC TGGCGCAGAG TGGAGCGGCC GCCGGAGATG

GTGXGAAGTT TGTXGTTGTT GTAGXGAGTT TGGXGTAGAG TGGAGXGGTX GTXGGAGATG

Zeschnigk PF

Kubota PR

C

CCTGACGCAT CTGTCTGAGG AGCGGTCAGT GACGCGATGG AGCGGGCAAG GTCAGCTGTG TTTGAXGTAT TTGTTTGAGG AGXGGTTAGT GAXGXGATGG AGXGGGTAAG GTTAGTTGTG

Exon I

Kosaki PR

Kosaki MR

Kubota MR

CCGGTGGCTT CTCTCAAGAG ACAGCCTGGG GAGCGGCCAC TTTTATTCAT CAGATATTCC

TXGGTGGTTT TTTTTAAGAG ATAGTTTGGG GAGXGGTTAT TTTTATTTAT TAGATATTTT

G

AAGTTTTTAG GACTTGGAGT ACTGAATAAA CGGAATTTGG GCCCTAAAGT CCTTTGTTCT

AAGTTTTTAG GATTTGGAGT ATTGAATAAA XGGAATTTGG GTTTTAAAGT TTTTTGTTTT

Zeschnigk common 3'primer

GGAGAACCAG ATCCGGAATG TTCAGAGGCT TGCTGTTGTG CCGTTCTGCC CCGATGGTAT

GGAGAATTAG ATTXGGAATG TTTAGAGGTT TGTTGTTGTG TXGTTTTGTT TXGATGGTAT

MLPA probe (4104-L4294)

CCTGTCCGCT CGCATTGGGG CGCGTCCCCC ATCCGCCCCC AACTGTGGTG TCGCGACAGG

TTTGTTXGTT XGTATTGGGG XGXGTTTTTT ATTXGTTTTT AATTGTGGTG TXGXGATAGG

TCCTATTGCG GGTGTCTGCG GTGGGAAGGG CGGTGGTGAC TGGGAGCATG C

TTTTATTGXG GGTGTTTGXG GTGGGAAGGG XGGTGGTGAT TGGGAGTATG T
